# Clinical and Sociodemographic Characteristics that May Affect Delays in Child Sexual Abuse Disclosures: Ten Years in Practice

**DOI:** 10.5152/eurasianjmed.2024.24441

**Published:** 2024-10-01

**Authors:** Hicran Doğru, İbrahim Selçuk Esin, Ahmet Nezih Kök, Onur Burak Dursun

**Affiliations:** 1 Department of Child and Adolescent Psychiatry, Atatürk University Faculty of Medicine, Erzurum, Türkiye; 2 Department of Forensic Medicine, Atatürk University Faculty of Medicine, Erzurum, Türkiye; 3 Department of Child and Adolescent Psychiatry, University of Health Sciences International School of Medicine, Istanbul, Türkiye

**Keywords:** Child sexual abuse, forensic medicine, offender, perpetrator, sexual abuse disclosure, victim

## Abstract

**Background:**

This study covers Child Sexual Abuse (CSA) survivors who had never disclosed the abuse until their psychiatric interview. There is a clear lack of understanding of which factors contribute to delays in the disclosure of CSA. Thus, we aimed to examine the risk factors and sociodemographic variables that may inhibit CSA disclosures.

**Methods:**

We retrospectively analyzed the files of patients who presented to a child and adolescent psychiatry clinic with different complaints between 2010 and 2020 in a tertiary hospital. All of these sexual assaults were officially reported by a child and adolescent psychiatrist to the competent authorities.

**Results:**

The mean period of time from abuse to disclosure was 28.4 ± 32.5 months. The time from abuse to disclosure was significantly negatively correlated with mothers’ education years (r = −.430, *P* < .01) and with the age of onset of sexual abuse (r = −.589, *P* < .001). Results of multiple linear regression showed that female sex and the earlier age of onset of sexual abuse were significant predictors of increased duration from abuse to disclosure.

**Conclusion:**

Our results provide insights into several risks that may affect the time from abuse to disclosure for CSA survivors. Studies with larger samples are needed to understand the factors affecting the time from abuse to disclosure.

**Main Points**

- Earlier age at onset of sexual abuse and female sex were associated with longer delays in disclosure.
- Lower maternal education was significantly associated with delays in CSA disclosures.
- Common psychiatric disorders in CSA survivors include conduct disorder, depression, and PTSD, but establishing causality remains challenging.

## Introduction

Child sexual abuse (CSA) is a global problem with many detrimental effects on individuals and society.^1^ A review of 55 studies reported prevalence estimates of child sexual abuse ranging from 3% to 17% for boys and 8% to 31% for girls.^2^ The estimates of child sexual abuse vary between 9% and 13% in Türkiye.^3,4^ Although, it is believed that CSAs are dramatically underreported because of cultural factors, nondisclosure, and the inability to recognize the wrongfulness of the act.^5^ An epidemiological study revealed that disclosure rates in childhood ranged from 31% to 41%.^6^ In addition, a recent study showed that 66.3% of children aged 10-17 years did not disclose the experience of CSA to parents or any adults.^7^

Child sexual abuse is a substantial risk factor for numerous psychiatric disorders, including post-traumatic stress disorder, sexual behavior problems, internalizing problems, insecure attachments, anxiety problems, and conduct disorders.^8^ More severe consequences are likely to occur when the victim delays or conceals the disclosure of CSA.^9^ Early access to supportive and therapeutic resources for CSA survivors can improve children’s mental health outcomes and psychosocial adjustment.^10^ Examining the psychosocial factors of children with delayed sexual abuse disclosures may help to understand the factors that contribute to this delay. Moreover, it can also help to prevent subsequent sexual victimization.

Many countries, including Türkiye, have laws regarding the obligation to report sexual abuse of children. All public servants, including medical professionals, are legally required to report suspected cases of child abuse.^11^ Türkiye imposes criminal and civil penalties on medical professionals who willfully fail to make a report when they suspect that a child is being sexually abused.^11^ Despite mandatory reporting laws, many officials do not always make a report when they suspect CSA for various reasons.^12^ One of the most common reasons for not reporting is not believing that the evidence for their suspicions is strong enough.^13^ Other reasons include sensitive socio-cultural or familial environments, inadequate education to identify clinical signs of CSA, and an inherent fear of difficulties expected to be encountered after a report.^14-15^ There are several ways to report child sexual abuse to the competent authorities. In Türkiye, a psychiatrist is obliged to report sexual abuse to the Public Prosecutor’s Office, child protection agencies, the police, or department of forensic medicine.^16^ However, the majority of Türkiye hospitals do not have a department of forensic medicine. In this study, an official reporting framework is used; relevant authorities in the forensic unit of a university hospital are informed about the incident and the cause for concern. A unit directed by the forensic medicine department is responsible for receiving and executing these statements. Through this unit, a communication channel functions between the reporting departments and the judicial authorities. The purpose of establishing this reporting framework system is to protect the privacy of the specialist who reports. Usually, information is disclosed directly to law enforcement by the family. Also, child survivors or their families want to disclose the sexual abuse. The psychiatrist becomes a part of this process after the official proceedings begin. Therefore, children who have been sexually abused are first evaluated in the department of forensic medicine and then referred to the department of child and adolescent psychiatry. Some of the survivors or their families do not want to disclose the sexual abuse, but it is discovered in some other manner. The psychiatrist is obliged to report this sexual abuse legally, even if the survivor and family do not want to disclose it.

Evidence from studies suggests that certain factors may impose a potential risk on delayed disclosures of CSA. These factors include being younger (because of a lack of knowledge), being victims of incest, male sex, feeling responsible for the CSA, lack of maternal support, type of abuse, fear of being blamed, and fear of the consequences of telling.^17,18^ Also, according to Finkelhor, the age-related impact of CSA differs between sexes; the rate of sexual abuse is lower among males aged 14-17 years compared to females.^19^ This result suggests that age may have an influence on delayed disclosures.

Findings from large-scale studies highlight the prevalence of delays in the disclosure. of CSA.^10^ Despite this, there are relatively a few data focusing on delayed disclosures of CSA and its outcomes.^20^ Here, we sought to examine the clinical and sociodemographic characteristics that may contribute to delays in CSA disclosures. We also sought to determine which factors are associated with the duration of time from abuse to disclosure in CSA survivors. We developed 3 hypotheses aligned with our study’s aim to identify the risk factors and sociodemographic variables contributing to delays in CSA disclosure. The hypotheses of the study are: (i) higher levels of maternal education are associated with shorter delays in the disclosure of Child Sexual Abuse (CSA), (ii) earlier age of onset of sexual abuse is associated with longer delays in the disclosure of CSA, and (iii) girls are more likely to experience delays in CSA disclosures than boys.

## Material and Methods

### Participants

This study consisted of file records of children and adolescents. All victims were formally reported by a child and adolescent psychiatrist. These file records are based on psychiatric interviews with these children and their families. Participants ranged from 9 to 17 years old at the time of the interview (Mean ± SD: 14.6 ± 2.2); they ranged from 6 to 17 years old at the time of the sexual assault (Mean ± SD: 12.5 ± 3.1). According to Finkelhor’s study,^19^ we compared males and females for CSA rates in the recommended age range (< 14, ≥14). Table 1 shows the socio-demographic characteristics of the victims.

Ethics committee approval was obtained from the Ethics Committee of Atatürk University (date: 07.05.2020, approval no.: 214).

### Data Collection and Analysis

This study was carried out at Atatürk University, one of the largest and oldest universities, located in the Eastern Anatolia Region of the Republic of Türkiye. This study is based on file records of 30 children and adolescents who were formally reported by a child and adolescent psychiatrist. These file records consisted of psychiatric interviews with both these children and their families. Children’s disclosure of sexual abuse was assessed through a review of official clinical records of cases processed from January 2010 to May 2020. Victims were interviewed with the Diagnostic and Statistical Manual of Mental Disorders, 4th Edition, Text Revision (DSM-IV-TR).

This study included a retrospective analysis of CSA survivors who had never disclosed the sexual abuse until their clinical psychiatric interview. The inclusion criteria of the study are: (i) children and adolescents who were capable of participating in an interview at the time of the psychiatric assessment; (ii) survivors of CSA who had never disclosed the abuse until their clinical psychiatric interview; (iii) victims who presented to the outpatient clinic with complaints other than sexual abuse; and (iv) cases that were officially reported to the authorities by a child and adolescent psychiatrist. However, this study excluded cases where the survivors had previously disclosed their experience of CSA to family members or friends before the psychiatric interview and cases with missing data essential for the study’s analysis. Complaints presented by the victims at their first interview, other than sexual abuse, are shown in Table 2. The data of 34 children with the above-mentioned characteristics were examined and 4 of the children were excluded. Exclusions were mainly due to missing data.

All children were evaluated in official reporting cooperation with the forensic medicine department. The services are provided jointly by the department of child and adolescent psychiatry and the department of forensic medicine after reporting. The final decision regarding the child is reported to the relevant authority, within the framework of the board decision. Psychiatric interviews and applied treatments or therapies are kept in an archive within the child psychiatry department.

We collected demographic and clinical data regarding victims and victims’ families. The victim’s age, sex, age at onset of sexual abuse, the time from CSA to disclosure, first clinical complaints of victims, number of sexual assaults, the location where the CSA took place, number of perpetrators, perpetrator’s relationship with the victim, victim’s self-harm or suicidal history, marital status of parents, economic status, and placement of residence after CSA (home/out-of-home care) were analyzed.

### Statistical Analyses

The data were analyzed using the Statistical Package for Social Sciences version 24 (IBM SPSS Corp.; Armonk, NY, USA). The normality of quantitative variables was tested by the Kolmogorov–Smirnov test. Categorical data were tested with the chi-square test and were given as numbers and percentages. Continuous variables were compared using the Student’s *t*-test. Correlations were assessed using Pearson’s correlation coefficient. Stepwise multiple regression analysis was used to determine independent factors affecting the duration from abuse to disclosure. Two-tailed statistical significance was accepted at *P* < .05.

## Results

A formal reporting framework was used in this study. Out of 30 children included in the study, 11 (37%) were males and 19 (63%) were females. All of these victims were severe cases of CSA involving vaginal or anal penetration. There was a significant difference between males and females in terms of mean age at the time of the interview [females (Mean ± SD): 15.4 ± 1.5, males (Mean ± SD): 13.1 ± 2.6; *P* = .005]. The time from the onset of the sexual abuse to the time of the first disclosure was considered as the delay in disclosure and ranged from 1 month to 120 months (Mean ± SD: 28.4 ± 32.5). Socio-demographic characteristics of victims are displayed in Table 1. Experienced sexual abuse was analyzed according to age and sex. The rate of experienced sexual abuse among females aged 14-17 was higher than among males of the same age, *P* = .029 (Table 1).

The most common places where CSA occurred were their own home (30%), schools (20%), and other places (50%) including other residential settings, school property, cars, streets, abandoned buildings, and hospitals (Table 2). The vast majority (90%) of victims were abused by someone they knew and trusted (relatives + acquaintances). Only 3 (10%) of the abusers were strangers (Table 3).

Six children were placed in out-of-home care after the disclosure of CSA. These children were sexually assaulted more than once by a family member (biological father or stepfather) (Table 4). Some of the victims attempted suicide and displayed self-harming behavior following the abuse. Descriptive analyses of study variables and behavior problems according to family status (nuclear family/divorced) were displayed in table 4.

The correlations revealed multiple significant associations among the variables and delays in disclosure between variables (Table 5). The duration from abuse to disclosure was significantly negatively correlated with the mothers’ education years (r= −.430, *P* < .01) and with the age of onset of sexual abuse (r= −.589, *P* < .001). Further, there was a negative correlation between the number of assaults and the duration from abuse to disclosure (r = −0.408, *P* < 0.05).

In order to exclude confounding factors, we selected sex, age of onset of sexual abuse, economic status, family status, mother’s education year, and number of sexual assaults as independent variables, and the duration from abuse to disclosure as a dependent variable in multiple stepwise regression analysis (Table 6). Ultimately, the equation suggested that the duration from abuse to disclosure was negatively correlated with the age of onset of sexual abuse and positively correlated with female sex (Adjusted *R* ^2^: 0.563, *F* (2,27) = 19.671, *P* < .001).

The duration of the follow-up period varied between 3 and 22 months (Mean ± SD: 10.07 ± 4.2). During the follow-up period, the children, common psychiatric problems among the children were as follows: Conduct disorder (n = 9), depression (n = 8), post-traumatic stress disorder (n = 5), attention-deficit and hyperactivity disorder (n = 4), obsessive-compulsive disorder (n = 2), panic disorder (n = 1), bipolar mood disorder (n = 1), schizophrenia (n = 1), sexual identity disorder (n = 1), anorexia nervosa (n = 1), enuresis (n = 1), and encopresis (n = 1). Five of the children had comorbid psychopathology.

## Discussion

The wide range of delays in the disclosure makes it difficult to obtain a reliable estimate of the prevalence of CSA. Approximately 1 in 4 females and 1 in 13 males experience CSA every year.^21^ A meta-analysis showed that females are at higher risk for CSA victimization than males.^22^ In this study, a total of 19 (67%) survivors were female. According to Finkelhor, males are more likely to be victimized than females at younger ages.^19^ Similarly, our study revealed that among victims younger than 14, boys were at a higher risk than girls of experiencing CSA (Table 1). These results may suggest that before the age of 14, boys are more vulnerable to CSA than girls. Further, we found a negative association between the duration from abuse to disclosure and the age of onset of sexual abuse and male sex. It can be argued that the prolonged period of duration from abuse to disclosure with decreasing age of onset of sexual abuse may be due to the lack of awareness of the child.

Remaining as neutral as possible during an interview of a child about the alleged abuse is the most important part of the assessment, as there are often no physical findings to justify the diagnosis.^23,24^ In the majority of cases with delayed official notification, a victim knows the abuser well.^25^ This familiarity is likely to trigger the secret-keeping behavior that the victims’ first complaints mainly consist of other psychological problems. In this study, 87% of the children presented with behavioral complaints and 90% were assaulted by someone they are familiar with. The time from abuse to disclosure was more than 2 years (Mean ± SD: 28.4 ± 32.5, months). This familiarity stands out as a factor that causes children to be easily manipulated during the incident and triggers delays in disclosure.^26^

There are some barriers (shame, guilt, lack of awareness, cultural factors, lack of family support, and the way the social environment reacts) to CSA disclosures for both males and females.^27,28^ Delaying in the disclosure of the assault was associated with a decreased age of onset of abuse (r = −.589). Relatively younger ages seem to be an obstacle to the child’s awareness of sexual abuse.^28^ Further, there was a negative correlation between the number of assaults and the time from abuse to disclosure. The child’s exposure to more than one assault may be a factor that facilitates CSA disclosure.

Although CSA influences all social levels, it is known that the risk of CSA is twice as high in lower socioeconomic status.^29^ In this study, children with delayed CSA disclosures were from families with lower-middle socioeconomic status and low education levels. Therefore, our findings represent families with low socioeconomic status. Moreover, the time from abuse to disclosure was negatively associated with mothers’ education years. This result is intriguing but may be a reflection of low socio-economic status. However, delayed disclosure of CSA may be associated with poor awareness, also contributed by low SES and low education levels. It is known that a low sociocultural level is a risk factor for child abuse.^30^ This seems to be an obstacle for child-raising awareness. Furthermore, mothers can play a critical role in the process from abuse to CSA disclosure. Indeed, a 17-year longitudinal study showed that one of the factors associated with the risk of child abuse was low maternal education.^31^ Lower maternal education levels might contribute to delays in CSA disclosure due to a lack of awareness and understanding of the signs of abuse. These mothers might not recognize the behavioral and emotional indicators of CSA, or they might attribute these signs to other causes. Additionally, they may lack knowledge about the appropriate steps to take when they suspect abuse, resulting in prolonged periods before seeking help. This gap in awareness and action can significantly delay the process of disclosure and the initiation of necessary interventions.

After the CSA disclosure, survivors with divorced parents were more likely to be placed in out-of-home care settings. Further, these survivors were more likely to have histories of self-harm. It is an undeniable fact that the nuclear family and the accompanying social climate are vital factors for subsequent stress and psychopathology.^32^ Parental backing to maintain the functioning of the child to the highest extent possible are rational and considerate responses and can help prevent the development of behavioral disorders.

The follow-up period allowed us to observe the emergence and progression of psychiatric symptoms and disorders post-disclosure, providing valuable insights into the mental health trajectories of CSA survivors. However, the relatively short follow-up period (Mean ± SD: 10.07 ± 4.2) may not capture the long-term psychological impact of CSA, limiting our understanding of the full spectrum of psychiatric outcomes.

Many studies have revealed that CSA survivors are at risk for suicide and psychological disorders such as post-traumatic stress disorder (PTSD), depression, social phobias, anxiety disorders, attention problems, and poor self-esteem, among which PTSD is the most common risk.^33-36^ In addition to these studies, conduct disorder was also a common diagnosis in this cohort. It is hard to know whether some of these disorders develop after CSA or act as a facilitator to the experience of CSA. Given that this study is based on a chart review of child sexual abuse disclosures, it is challenging to determine whether psychiatric disorders developed as a consequence of the abuse or were pre-existing conditions. Thus, establishing a clear relationship between these disorders and CSA remains difficult.

Our study faced several limitations, including a small sample size and potential biases. Given the rarity of psychiatrist disclosures in our center, our cohort represents only a small, selected subset of victims, which may limit the generalizability of our findings. Additionally, our study was confined to a clinical sample of victims interviewed at a single center, predominantly from low socioeconomic backgrounds. The presence of potential biases, such as selection or reporting biases, may have influenced our outcomes. Acknowledging these limitations is essential for a more nuanced interpretation of our results and highlights areas for further research.

The study underscores the necessity for larger, multicenter studies to validate these findings and explore additional factors influencing the timing of CSA disclosures. Such research could aid in the development of targeted interventions and policies aimed at reducing delays in disclosure., thereby improving outcomes for CSA survivors. Future studies should also examine the role of maternal education in greater detail to understand its specific impact on disclosure timing and develop effective educational programs for parents.

Recognizing the significant impact of socioeconomic factors, such as maternal education level, on the timing of CSA disclosures underscores the importance of tailored interventions targeting families, especially those from disadvantaged socioeconomic backgrounds. Early identification and intervention strategies aimed at facilitating disclosure and providing support services can mitigate the adverse effects of CSA and promote healing and resilience in survivors. Policymakers should prioritize funding and resources for multifaceted prevention efforts, including education programs targeting parents, caregivers, and communities to raise awareness about CSA and enhance protective factors. Additionally, there is a critical need for policies that ensure timely access to mental health services and support for CSA survivors, particularly those from vulnerable backgrounds who may face additional barriers to care.

Our study identified several key factors influencing delays in CSA disclosure. The results underscore that age at onset of sexual abuse and sex were independently associated with the duration from abuse to disclosure. Specifically, younger age at onset and female sex were significant predictors of longer delays. Additionally, lower maternal education levels were significantly associated with longer delays in disclosure., highlighting the critical role that mothers play in recognizing and responding to CSA. This relationship suggests that maternal education can enhance awareness and promptness in addressing CSA, independent of broader socioeconomic factors.

Common psychiatric disorders observed in our cohort included conduct disorder, depression, and PTSD. However, due to the retrospective nature of the study, it remains difficult to ascertain whether these disorders were a consequence of CSA or pre-existing conditions.

Given these findings, early interventions and efforts to raise awareness, particularly among mothers with lower education levels, are crucial to preventing delays in CSA disclosure. Further, larger-scale studies are necessary to explore the variables associated with delays in disclosure. more comprehensively and to develop strategies for more effective intervention.

## Figures and Tables

**Table 1.** Socio-Demographic Characteristics of the Victims

	n	%	Mean ± SD
Males	11	37	
Females	19	63	
Duration of Education Year of All Victims			8.3 ±1.8
	Male	Female	*P*
Age groups at onset of sexual abuse, n (%)			
6-13 years	8 (73)	6 (32)	**.029**
14-17 years	3 (27)	13 (68)	
Age at onset of abuse, mean ± SD	12 ± 3.1	13.4 ± 3.0	.248
Age at the time of the interview, mean ± SD	13.1 ± 2.6	15.4 ± 1.5	**.005**
Time from the onset of the SA to the time of the first disclosure (months), mean ± SD	19.2 ± 20.8	33.8 ± 37.2	.243
Child-on-child sexual abuse, n (%)	5 (45)	–	**.001**
History of self-harm, n (%)	3 (27)	14 (82)	**.023**

Values in bold indicate statistical significance.

**Table 2.** Socio-Demographic Characteristics of Victims

	n	%	
Socio-Demographic Details of Parents			
Mothers age, mean ± SD			42.4 ± 7.4
Fathers age, mean ± SD			46.1 ± 7.6
Mother’s education year, mean ± SD			4.5 ± 3.2
Father’s education year, mean ± SD			8.9 ± 3.6
Number of Siblings, Mean ± SD			4.2 ± 1.9
Marital status of parents			
Divorced	9	30	
Married	21	70	
Economic Status of the Family			
Minimum wage or less income	21	70	
Medium	9	30	
Abuser’s Relationship with the Child			
Relative	8	27	
Acquaintance	19	63	
Stranger	3	10	
Place of abuse			
Own house	9	30	
School	6	20	
Other	15	50	
Notification Time			
First interview	25	83	
Subsequent interviews	5	17	
Placement of Residence After CSA			
Own home (with family)	24	80	
Out-of-home care	6	20	

**Table 3.** First Complaints of Patients

	n	%
Medical complaints (total)	3	10
Syncope	1	3.3
Enuresis	1	3.3
Encopresis	1	3.3
Application for health report	1	3.3
Behavioral changes (total)	26	87
Inattentiveness	1	3.3
Hand washing	1	3.3
Aggression	3	10
Learning disability	1	3.3
Perception problem	2	6.7
Resorting to lies	1	3.3
Thoughtfulness	1	3.3
Depression	2	6.7
Hyperactivity	1	3.3
Escaping from school	1	3.3
Fear	1	3.3
Suicidal ideation	2	6.7
Explicit description of sexual contact	5	16.7
Sexual perpetration on others	1	3.3
Compulsive masturbation	3	10

**Table 4.** Characteristics of the Groups According to Marital Status of Parents

	Married		Divorced		*t*/*Χ* ^2^	*P*
	(n = 21)	%	(n = 9)	%		
Age of victims, (mean ± SD)	14.7 ± 2.0		14.3 ± 2.7		0.475^a^	.639
Age at onset of sexual abuse, (mean ± SD)	12.7 ± 3.1		12.3 ± 3.2		0.306^a^	.762
Sex, (male/female)	10/11	48/52	1/8	11/89	3.616^b^	.057
History of self-harm, (yes/no)	9/12	43/57	8/1	89/11	5.436^b^	**.005**
Suicide attempt, (yes/no)	1/20	5/95	2/7	22/78	2.134^b^	.144
Number of sexual assaults, (once/more than once)	8/13	38/62	4/5	44/56	0.106^b^	.745
Placement of residence (home/out-of-home care)	19/2	91/9	5/4	56/44	4.802^b^	**.028**
Age of mothers, (mean± SD)	42.5 ± 7.8		42.1 ± 6.0		0.111^a^	.913
Age of fathers, (mean± SD)	46.6 ± 7.8		42.1 ± 4.2		0.807^a^	.431
Mother’s education year (mean± SD)	5.2 ± 3.5		4.0 ± 2.4		0.940^a^	.355
Father’s education year (mean± SD)	9.1 ± 3.7		8.3 ± 3.4		0.526^a^	.603
Economic status of the family (minimum wage/medium)	13/8	62/38	8/1	89/11	2.184	.139

^a^Student’s *t-*test.

^b^ Chi-square test.

**Table 5.** Bivariate Correlations Analysis Between Variables

	1	2	3	4	5	6	7
1. Age at onset of sexual abuse	–						
2. Economic status	−.400*						
3. Number of assaults	.395*	−.208	–				
4. Number of abusers	.319	−.029	.764***	–			
5. Mother’s education year	.359	−.040	−.097	−.168	–		
6. Father’s education year	−.114	.169	−.012	−.040	.348	–	
7. Duration from abuse to disclosure	−.589***	.359	−.408*	.215	−.430**	−.143	–

*P < .05.

**P < .01.

***P < .001.

**Table 6.** Multiple Stepwise Regression Analysis Showing Variables Independently Associated with Changes in the Duration from Abuse to Disclosure

	*B*	SE *B*	*β*	*t*	P
Age at onset of sexual abuse	−7.891	1.313	−0.747	−6.011	**<.001**
Sex (male,female)	22.083	8.250	0.332	2.667	**.012**

A multiple stepwise regression analysis was carried out. (Adjusted *R* ^2^: 0.563, *F* (2,27) = 19.671, *P* < 0.001).

SE *B*, standard error of the mean.

## Data Availability

The data that support the findings of this study are available on request from the corresponding author.
